# The effect of trainee career intentions on mentor’s interest in the trainee: Experimental evidence from academia

**DOI:** 10.1016/j.respol.2025.105232

**Published:** 2025-03-20

**Authors:** Inna Smirnova, Austin Shannon, Misha Teplitskiy

**Affiliations:** a University of Michigan School of Information, Ann Arbor, MI 48109, United States of America; b Michigan Institute for Clinical and Health Research, Ann Arbor, MI 48109, United States of America; c Department of Microbiology and Immunology, University of Michigan, Ann Arbor, MI 48109, United States of America

**Keywords:** Academic labor market, Selection of trainees, Mentor-trainee career mismatches, Audit experiment

## Abstract

In many industries trainees often seek careers different from their mentors. For example, many PhD students seek non-academic careers. Anecdotally, mentors invest less in different-career trainees, but causal evidence is lacking. To fill this gap, we conducted an audit experiment in academia, where a fictitious prospective PhD student emailed immunology and microbiology principal investigators (PIs) about mentorship. The student’s career intention was randomly described as “applied research in industry” (*n* = 1000), “basic research in academia” (*n* = 1000) or no description (control, *n* = 442). To mitigate concerns about skills and motivation, all emails highlighted the student’s great academic record. Contrary to expectations, PIs responded at similar rates across all conditions. Treatment effects showed little heterogeneity based on the PIs’ institution prestige, industry connections, and career length. These null findings challenge the widespread belief that mismatched career intentions *cause* less mentorship (although the two may still be associated) and the mechanisms assumed to drive that effect. Our results call for caution in deploying interventions to fix problems related to advisor-mentee misalignments before clearly establishing their source.

## 1. Introduction

Many industries rely on an apprenticeship model in which a mentor helps a trainee master skills over time (Lerman and Rauner, 2012). The model is used in industries ranging from construction and manufacturing, to entrepreneurship and medicine, and to the arts and doctoral education (Anderson and Gold, 2019; Slavich and Castellucci, 2016; Smyth and Zimba, 2019). The satisfaction and attrition rates of trainees in apprenticeship vary substantially (Gambin and Hogarth, 2016; Keloharju et al., 2024; Ragins et al., 2000; Smyth and Zimba, 2019; Snell and Hart, 2008; Ullrich et al., 2021; Zimmerman, 2018). Factors linked to attrition include poor working conditions, insufficient training, personal circumstances, and external labor market conditions (Gambin and Hogarth, 2016; Ysseldyk et al., 2019). Combining these factors with the high levels of competition in apprenticeship-focused industries like the arts and academia (Clauset et al., 2015; Slavich and Castellucci, 2016), it is not surprising that trainees often prefer not to (Fuhrmann et al., 2011; Mason et al., 2009; Roach and Sauermann, 2010; Sherman et al., 2021) or are unable to pursue the same career as their mentor (Check Hayden, 2016; Cyranoski et al., 2011; De Janasz et al., 2003; Ganguli et al., 2022). This in turn results in the abundance of mismatches between mentors’ careers and their trainees’ career intentions across industries (e.g., Eby et al., 2004). Indeed, in areas like the life and health sciences most PhD graduates pursue non-academic careers (Langin, 2019), so mismatches are the rule rather than the exception (Fuhrmann et al., 2011).

A voluminous literature points to an association between career mismatches and negative consequences. For instance, mentors tend to provide more career development and psychosocial support to trainees more similar to themselves in background and ambition (Burke et al., 1993; Limeri et al., 2019; St. Clair et al., 2017). With regards to the applicants’ explicit career intentions, the existing literature is limited to survey explorations only. For example, a U.S. National Institutes of Health (NIH) survey of graduate and postdoctoral trainees found that matching career interests between a trainee and a principal investigator (PI) are associated with higher levels of trainee career self-efficacy (Chatterjee et al., 2023). Similarly, a survey of U.S. biomedical postdocs by Gibbs et al. (2015) reported that only half of the participants mentioned that their graduate advisors supported non-academic career paths, while fewer than one-third agreed that their departments were supportive of students interested in other career paths. As a result, trainees with mismatched intentions often report experiencing psychological distress, feeling “afraid of being judged—or worse, ostracized—by faculty who are not supportive of exploring alternative careers during PhD training” (Schillebeeckx et al., 2013). The association may be driven by several causal mechanisms, foremost of which is mentor’s self-interest: trainees may provide more long-term benefits like prestige to mentors when they stay in the same industry (Sherman et al., 2021). If career mismatches cause less or lower-quality mentorship and this is well known, it could affect not only existing mentorship relationships but selection into the career—only those very confident that they will place into the mentor’s career will select into the path. Such self-selection may ultimately hurt trainees’ satisfaction and cause the field to lose out on “Einsteins” (Bell et al., 2019) who never even applied.^1^ The severity of these negative experiences has led to many calls for mitigation strategies (Williams et al., 2016), such as encouraging or training mentors to better support *all* trainees (Agarwal and Sonka, 2010; Gruber et al., 2024; Langin, 2019; Lenzi et al., 2020; Thiry et al., 2015).

Yet despite the ubiquity of mismatches, their seemingly negative consequences, and plausible causal mechanisms, the evidence base has two fundamental limitations. First, the question of how career mismatches affect mentorship is fundamentally causal but, to our knowledge, all evidence in this literature is associational. Consequently, it is possible that the observed associations are due to reverse causation, such that poor mentorship causes trainees to choose alternative careers, or confounding, such that low commitment or another factor causes both less mentorship and alternative career choice. Second, nearly all existing studies rely on self-reported outcomes, which may be biased due to social desirability or other reasons. There is thus a gap in evidence regarding causality and mechanisms connecting mentor-mentee mismatches and behavioral outcomes like selection. Filling this gap would help inform mitigation strategies that could target specific mechanisms causing negative consequences for mentors and mentees.

This study fills the gap by posing and answering the following research question—Does a trainee’s career intention *causally* affect mentors’ responses to prospective trainees? Drawing on the literature streams on human capital, social capital, and mentor’s self-concept, we hypothesize that mentors will select against mismatching trainees. Additionally, we explore heterogeneity across mentors, focusing on their connections outside of their own industry, career length, and organizational prestige.

We study the setting of academic life sciences, given the prominence of the apprenticeship model and the substantial and consequential discretion exercised by mentors (Anderson and Gold, 2019; Milkman et al., 2015). Prior research has reported that entry into academia is highly competitive (Clauset et al., 2015; Milojević et al., 2018; Van Bavel, 2019), with a large number of doctorate recipients working in the private sector (Opsomer et al., 2021). In the life sciences, the numbers are especially high given the field’s lack of expansion and the resulting mismatch between supply and demand (Alberts et al., 2014). Statistics show that fewer than 20 % of PhD recipients end up in tenure-track positions within 5–6 years after degree completion (Stephan, 2015). Nevertheless, a PhD and postdoctoral training are often required for advancement not only in academia but also to senior roles in the private and non-profit sectors. The field thus consistently remains in the leading position in the amount of PhD degrees it awards, with a steady increase over the past two decades (Langin, 2019; National Center for Science and Engineering Statistics, 2023). We focus on entry into a profession—the steps *before* an individual formally applies to an opening, such as informal interactions (Lane et al., 2023; Milkman et al., 2012, 2015; Posselt, 2016; Rivera and Tilcsik, 2023). These early steps can be determinative for a prospective trainee’s choice to actually enter the profession, affecting their entire subsequent career. For mentors, selecting whom to mentor is equally consequential. First, for mentors who provide funding for their trainees, the financial stakes are high. Second, in many PhD programs the mentor is effectively “locked in” to a particular trainee until graduation, making it a multi-year commitment. Consequently, if career mismatches causally affect mentorship, the effects should appear at this stage, where mentors have substantial discretion and stakes.

We performed an audit experiment with 2442 principal researchers in the fields of microbiology and immunology^2^ who received emails from a fictitious prospective PhD student requesting to discuss mentorship opportunities before applying to a PhD program. Audit experiments entail randomizing information provided to participants, for example randomizing the name on resumes sent to employers, and have become among the most widely used and trusted methods for studying discrimination in the labor market (Neumark, 2018). They are used to establish the causal effects of the information on behavior, typically reply rates. Prior work has found that even small changes in the information provided, such as the name and affiliation of an email sender (Ajzenman et al., 2023; Buzard et al., 2023; Milkman et al., 2015; Rivera and Tilcsik, 2023) or whether a meeting is requested for “today” or “next Monday” (Milkman et al., 2012), can have substantial effects on participants’ response. In our study, we randomly assigned PIs to receive an email that described the prospective student’s career interest as either “*applied research in industry*” or “*basic research in academia*” or no description (control group).

We found that PIs were generally less responsive in the industry condition but the effect was only weakly significant and not robust to all specifications. We are thus unable to reject the null hypothesis that PIs were similarly responsive to students in all conditions. The content analysis of the received responses confirmed this finding. In the additional analyses, we explored the heterogeneity across mentors’ characteristics and whether it moderates the selection decisions, hypothesizing that selection against trainees with industry career aspirations would be largest by mentors affiliated with more prestigious institutions and with fewer connections to industry, and directionally unclear in terms of mentor seniority. We found, however, that the treatment effects were similar across all PIs’ characteristics.

Our study contributes to the understanding of scientific labor markets and general labor market discrimination by going beyond the demographic characteristics of applicants. Instead, focusing on their stated career intentions, we provide first causal evidence on their effects. Practically, our findings paint an arguably positive picture—in the academic life sciences, mentors do not discriminate against industry-leaning prospective PhD students, at the point of entry into the profession. Conceptually, this challenges the widespread view that mismatches *cause* lower mentorship. Relatedly, our experimental design and results challenge the assumed mechanism that a trainee’s same-industry career is in mentor’s self-interest, and that this will dominate their decision-making around mentoring.

If the relationship between a trainee’s career intention and lower mentorship availability as often reported in surveys is not causal, what could explain it? Several possibilities might play a role, including: 1) reverse causation, 2) the scope condition of a trainee’s academic skills (that are kept constant in our design) being the primary concern driving mentors’ selection decisions, and 3) the long-term consequences of career mismatch being of minor importance or even desirable for mentors who want to increase their social capital. We return to these possibilities in the Discussion section.

## 2. Theoretical background

### 2.1. Mentor-trainee career mismatches and the availability of mentorship

#### 2.1.1. Human capital: Trainee’s skills and commitment

Organizations typically evaluate candidates for competency and commitment to the organization (Rivera, 2020). The evaluation usually entails significant uncertainty and time constraints, so employers often rely on observable signals of skills such as education and work experience (Bidwell et al., 2015; Leung, 2014). Employers also pay attention to signals in a candidate’s profile to infer their fit, i.e., cultural, and long-term commitment to an organization (Botelho and Chang, 2023; Galperin et al., 2020; Rivera, 2012; Weisshaar, 2018). These considerations should also apply to the selection of trainees by mentors, since mentors are likely to prefer highly skilled and committed trainees.

A strand of research on scientific career preferences suggests that academic scientists have a disproportionately strong “taste for science” (Janger and Nowotny, 2016; Roach and Sauermann, 2010) which is taken to mean intellectual freedom and challenge, and engagement with the scientific community by publishing. A trainee’s industry career interest will result in the mismatch between the mentor’s and trainee’s respective “taste for science” as industrial research is different from academic research, both in terms of process and output, resulting in a different skill set and motivations required to be productive (Agarwal and Ohyama, 2013; Roach and Sauermann, 2010; Sauermann and Roach, 2014). Consequently, students with non-academic career aspirations will be likely perceived as having different human capital, presenting lower payoff for the mentor’s and department’s academic environment and reputation (Roach and Sauermann, 2010).

A related strand of research on selections in science shows that when evaluating quality of prospective graduate students, committees do not only prioritize objective quantifiable achievements like high test scores but also value more subjective factors such as research fit (Green and Bauer, 1995; Posselt, 2016). Given the differences between academic and industrial research, student’s demonstrated career interest in industry will signal poor research fit and lower overall commitment to the field of academia (Sauermann and Roach, 2014), leading to concerns about student’s leaving the PhD program prematurely.

Together, PIs’ perceiving industry-oriented trainees of having different skills and motivations, and lacking commitment to perform academic research would predict that students with non-matching (industry vs. academia) career interests will be selected against.

#### 2.1.2. Social capital

Social capital refers to “the sum of the actual and potential resources embedded within, available through, and derived from the network of relationships possessed by an individual” (Nahapiet and Ghoshal, 1998). Social capital can be of great importance in academia, given the prevalence of teamwork and interdependencies among researchers (e.g., Gonzalez-Brambila et al., 2013; Khanna, 2021; Malmgren et al., 2010; McFadyen and Cannella, 2004; Zhang et al., 2022). For example, prior work shows that institutional connections to researchers with specialized knowledge can improve performance of their colleagues (Kolympiris et al., 2019), particularly junior ones (Hoenen and Kolympiris, 2020; Slavova et al., 2016). Similarly, research documents the significant impact of the “chaperone effect” on the inexperienced scientists’ achieving higher status in scientific publishing (Sekara et al., 2018). Given the substantial investment, trainees can become an essential part of the mentors’ social capital, potentially providing access to external resources after, and maybe even during, their training.

On the one hand, trainees with academic career intentions bring a direct social capital benefit to the mentor, both during and beyond the training period. Surveys of faculty advisors by Sherman et al. (2021) found that faculty often prefer their students to stay in academia to keep connected—“I like it when they go into research positions because it means I’ll continue to see them regularly at conferences—I like my students and having them move into career paths where I likely will not see them again is a personal loss for me—but that is grounded in the deepest and narrowest of my selfish desire to remain connected.” The loss of these connections could weaken the mentor’s network, affecting their access to resources and thus performance downstream. One concrete way in which academic social capital can affect mentors’ performance is that scientific impact requires not only high-quality work but a “sales force” to promote that work (Azoulay et al., 2019) as researchers do not have full awareness of all relevant scientific works and certainly no time to read them all (Simcoe and Waguespack, 2010). In practice that sales force is often composed of former trainees or other associates. Hence, the more a PI’s trainees stay in academia and publish, the larger the PI’s sales network and personal utility gains, benefiting them even long after the training period (Breneman, 1976). Choosing trainees with non-academic career aspirations will thus be a suboptimal decision for the mentor’s utility from mentoring.

On the other hand, having former trainees with non-academic careers can help expand a mentor’s professional network to a different industry. The expanded, broader network typically offers valuable resources such as opportunities for funding and collaboration, access to non-redundant information, and professional engagement (e.g., consulting, job rotations, or invitations to give talks), enhancing a mentor’s influence in the scientific community and thus helping advance their career prospects (Hayter and Parker, 2019). This perspective would suggest that selecting trainees with non-matching career intentions might not be as detrimental as initially presumed but rather beneficial for the mentor. Although a possible path for network and resource expansion, it requires trainees to first graduate and then successfully build careers in a different industry outside academia, thus implying significant delays in the benefit that the mentor can enjoy. We thus believe this potential benefit will be a second-order consideration for mentors when selecting whom to mentor, and will likely not manifest itself strongly at this early stage.

#### 2.1.3. Mentor’s self-concept

A strand of psychology research on identity and self-concept reinforces the mentor’s self-interest argument—some mentors might see an industry interest as a challenge to their own positive self-concept (Bailey, 2003; Dunning et al., 2004; Sherman and Cohen, 2006; Swann Jr. and Bosson, 2010). Specifically, a trainee’s different-industry career interest (partially) undermines the mentor’s view that their choice of academia is highly desirable and uniquely prestigious. 26 % of PhD students interviewed by Leong et al. (2024) reported that their advisor holds this view, being “very open in his dislike for the private sector, and his belief that academic positions are generally superior in every way.” The mentor’s desire to keep their positive self-concept intact will thus prevent them from selecting trainees with non-matching career interests.

Further threatening the mentor’s self-concept, industry-oriented trainees are more challenging to advise if the mentor is lacking knowledge of the trainee’s non-matching industry (Agarwal and Ohyama, 2013; Golde, 2005; Hayter and Parker, 2019). The survey study of PhD advisors by Leong et al. (2024) reported that 14 % of the respondents explicitly mentioned that they “know very little about nonacademic careers, so (they) struggle to provide adequate mentorship for a student who wants to pursue that path.” It is possible that some PIs who are motivated by challenges may prefer to invest in building human capital that would allow them to effectively mentor industry-oriented students without hurting their positive self-concept. This investment is risky and resource-demanding, however, given the trade-off researchers face when allocating their time to different aspects of their work. We thus expect that only a fraction of PIs would be willing to make such investment when selecting whom to mentor.

Each of these mechanisms independently points to the following prediction:

**Hypothesis 1**. **(H1)**. Prospective trainees with industry career aspirations will receive fewer responses than academia-oriented trainees.

### 2.2. Moderating the baseline: Mentor’s industry connections, seniority, and institutional prestige

After establishing the baseline prediction between a trainee’s career interest and a mentor’s interest in selecting the trainee, we consider heterogeneity across mentors. We focus on three mentor characteristics that may moderate their responses to industry- vs. academia-oriented trainees: 1) their existing ties to industry, 2) career stage, and 3) prestige of the institution where they are employed.

#### 2.2.1. Industry connections

There are substantial efforts across the globe to encourage universities to commercialize the research of their faculty through patent licensing and similar means (Ambos et al., 2008; Argyres and Liebeskind, 1998; Larsen, 2011). The biomedical field has received special attention as innovation in this field has long been described as a product of researchers’ co-mingling in both scientific and industrial networks (Murray, 2002). It has thus become common to see biomedical scientists have industry connections via being affiliated with firms directly or having collaborations with industrial scientists (Azoulay et al., 2007; Bikard et al., 2019; Lacetera, 2009; Perkmann et al., 2019). For example, in a sample of 2294 life science researchers, Haeussler and Colyvas (2011) found that 47 % of researchers were involved with industry through consulting, patenting, or company founding. Relatedly, prior experience and engagement with work outside academia has been associated with a more positive attitude towards industry (Van Dierdonck et al., 1990) and a higher willingness to collaborate with industry partners (Tartari et al., 2012).

We argue that, all else held constant, having industry connections should make PIs more accepting of industry-interested trainees. First, they will have a closer knowledge of industry, and thus better ability to mentor such trainees and help them find work. Second, PIs with industry connections will also personally benefit more from mentees working in industry, as industry-placed mentees will further strengthen their position in and ties to the industrial world and resources available through them. Lastly, PIs with industry connections may over time develop a stronger taste for commercialization and simply become more favorable towards industrial research and, possibly, employment (Hayter et al., 2022; Roach and Sauermann, 2010). This shift might be driven by either selection or professional imprinting. Specifically, the literature on imprinting (Marquis and Tilcsik, 2013) shows that certain career experiences—such as organizational culture or mentors and coworkers—can have long-lasting effects (Azoulay et al., 2017; Dalziel and Basir, 2024; Dokko et al., 2009; Higgins, 2005; Mathias et al., 2015). For example, in science, Azoulay et al. (2017) demonstrated that postdoctoral trainees in the biomedical field adopt their advisers’ orientations towards commercial science in their post-training careers (similar result found in Roche (2023)). In the field of biotechnology, Aschhoff and Grimpe (2014) similarly found that a scientist’s involvement with industry increases if they have co-authors who are industry-oriented. Building on these findings, we expect that PIs with industry connections will be more engaged with industry and thus more willing to take in industry-oriented trainees.

Taken together, we hypothesize:

**Hypothesis 2**. **(H2)**. Prospective trainees with industry career aspirations will be favored more by mentors with industry connections than those without.

#### 2.2.2. Seniority

Career length of a mentor may also affect their interest in industry-interested trainees. Studies have repeatedly demonstrated that mentors with experience differ from the inexperienced mentors with regards to their anticipated costs and benefits of mentorship (Ragins and Scandura, 1999). Relatedly, research has shown that junior and senior researchers have different priorities for their jobs. Based on the results from the large-scale quasi-experiment by Janger and Nowotny (2016) early-stage researchers especially value their research freedom and are “willing to pay” for it, while later-stage researchers favor jobs which help increase their income.

Research has also examined the general attitudes of junior and senior scientists towards collaborating with industry and doing more applied research, reporting mixed results. For example, Bercovitz and Feldman (2008) found that the longer the elapsed time since a faculty member’s graduation, the less likely they are to support research commercialization activities. Schuelke-Leech (2013), on the contrary, found a positive association between having tenure and the greater number of years since earning a PhD and an individual’s involvement with industry in a sample of U.S.-based STEM researchers (similar results in Haeussler and Colyvas (2011)). Ding and Choi (2011)’s study on a sample of U.S. life scientists showed a more complex picture—the likelihood of getting involved in company founding activity happens much earlier than that of advising a company activity in a scientist’s career—suggesting that the association is not linear and depends on the type of involvement with industry.

The relationship between the researchers’ academic age and their attitudes towards post-PhD careers in industry has not been established, however. The competition and imbalance between the supply and demand in the academic labor market have intensified in the last decade and continue growing (Larson et al., 2014; Sauermann and Roach, 2016). More junior faculty members who have graduated not long ago and managed to secure academic appointments should be more familiar with these challenges. This in turn can make them more accepting of alternative, non-academic career paths and view career outcomes as decreasingly informative about trainee competence to perform research. At the same time, research has shown that researchers who are more central within the academic research system are likelier to have connections to the industrial world (Giuliani et al., 2010), and centrality and social capital increase with a researcher’s academic age. It is thus possible that senior researchers will have more positive attitudes towards industry careers, on average.

Together, it remains unclear what direction of the relationship will be more prevalent at the trainee selection stage, and we thus aim to test two alternative predictions:

**Hypothesis 3a**. **(H3a)**. Prospective trainees with industry career aspirations will be favored more by more junior mentors.

**Hypothesis 3b**. **(H3b)**. Prospective trainees with industry career aspirations will be favored more by more senior mentors.

#### 2.2.3. Institutional prestige

Lastly, we argue that the prestige of a mentor’s institution will also affect how they respond to academia- vs. industry-oriented students. Prestigious institutions usually expect their faculty to focus primarily on research, enabling high publication and citation rates (Long, 1978; Way et al., 2019). As a result, scientists working at elite institutions rarely exit university research careers compared to their colleagues at less-prestigious universities (Geuna and Shibayama, 2015). Institutional prestige has also been negatively associated with the amount of interactions that the researchers have with the private sector (D’Este and Patel, 2007; Ponomariov, 2008), reinforcing their focus on academic research solely. Prestigious-institution scientists publish at higher rates than others and depend on graduate and postdoctoral labor to do so, particularly in lab-based fields like the life sciences (Zhang et al., 2022). We expect such scientists to value the focus on research and academic publishing especially highly and have less exposure to non-academic work, and thus seek trainees with the same goals. In addition, prestige of an institution is highly related to the *academic* placement of its PhD graduates (Burris, 2004), and mentors in these institutions will likely want them to remain prestigious.

These considerations lead to the following hypothesis:

**Hypothesis 4**. **(H4)**. Prospective trainees with industry career aspirations will be favored less by mentors from more prestigious institutions.

**Fig. 1.**
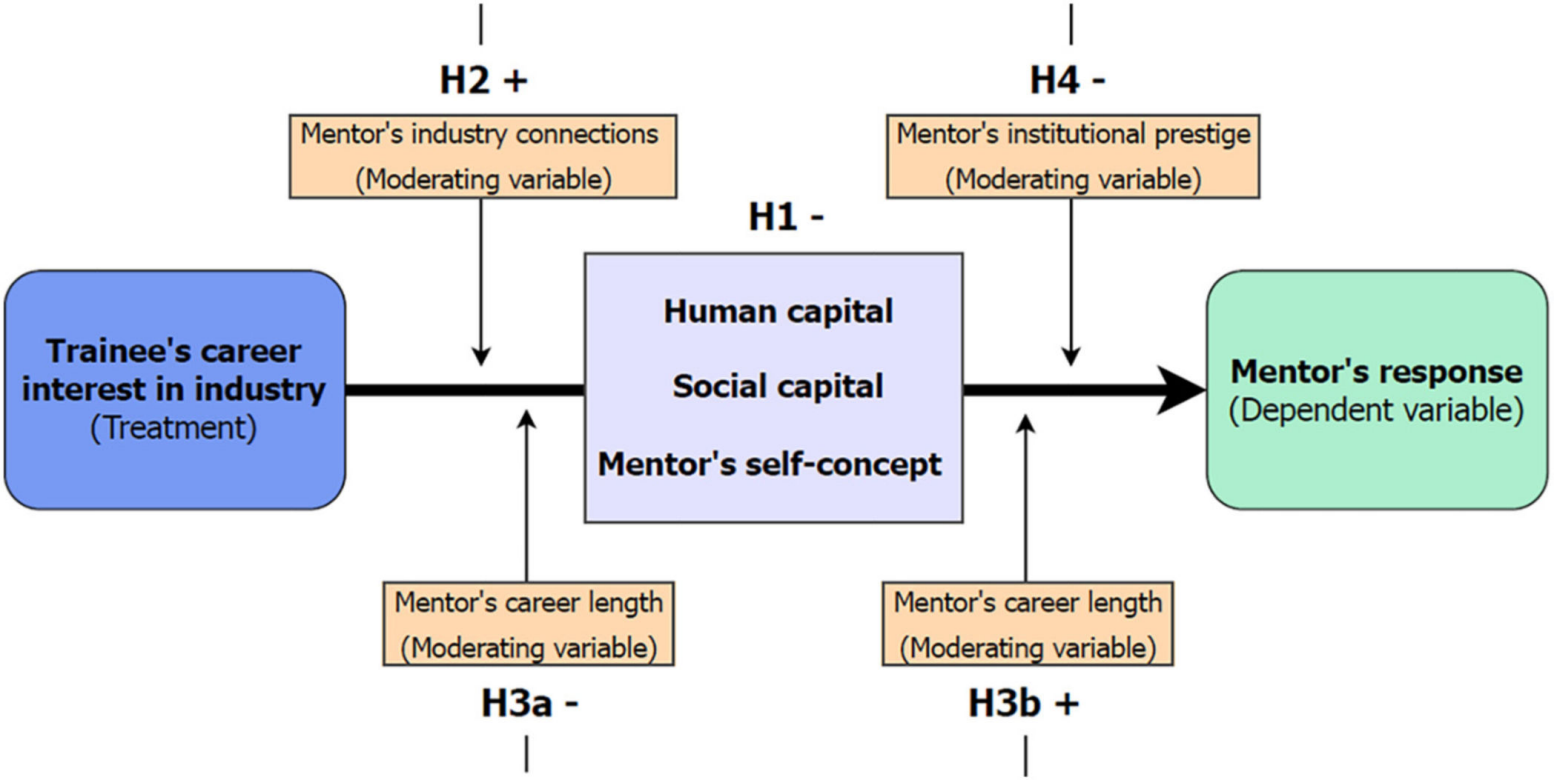
Overview of the paper’s conceptual framework and hypotheses.

Our conceptual framework and derived hypotheses are summarized in Fig. 1.

## 3. Research design, data, and methods

We use the audit study design to causally test whether signaling one’s interest in a future career in industry vs. academia affects the likelihood that a prospective PhD student will receive a response from a PI regarding their work and possible mentorship. We focus on the setting of academic life sciences. Empirically, the life sciences field is characterized by an increasing tendency of PhD holders to transition to non-academic careers, often involving research in the private sector. Recent work has emphasized the importance of close interest overlap between the protégé and their faculty mentor in predicting the odds of mentees’ continuing in academia (Liénard et al., 2018; Ullrich et al., 2021). Academia is also a setting with accumulating qualitative evidence that trainees’ career choices affect mentors’ advising, investments, and longer-term career outcomes (Boeder et al., 2021; Malmgren et al., 2010). Furthermore, the life sciences and academia more generally are an attractive site because academic mentors have a strong “taste for science” (Roach and Sauermann, 2010) and thus may care about trainee’s *commitment to the field of academia* as trainee’s career and research output affect their own reputation.

Our experiment was approved by the University of Michigan’s Institutional Review Board (IRB) protocol number HUM00225526. The audit method includes several advantages such as its ability to provide more direct causal evidence than archival studies and its realism compared to surveys and laboratory experiments (Couture, 2008; Rivera and Tilcsik, 2023). Our research design builds on several existing audit experiments with academics (Ajzenman et al., 2023; Angeli et al., 2023; Milkman et al., 2012, 2015), and uses a between-subjects design with three arms, where the emails presented a diligent student on the cusp of graduation with research experience and specific interest in the work of the email recipient. The singular difference in each of these emails was the stated future career interests. The control condition (email) had no stated future career interest, while the other two either indicated interest in academia or industry. Sending one rather than multiple emails to each participant helped mitigate ethical concerns and inconvenience to our subjects, as well as minimize the likelihood of spillovers and suspicion. Neither the fictitious student nor the experimenters responded to any replies to the initial email. We screened the replies received for any sign of a researcher recognizing that the email was sent from a fictitious student and that the participant might be part of an audit experiment. None of the screened replies and systematic searches of all replies with keywords like “experiment” or “audit” indicated such concern.

In consultation with the IRB and in line with prior similar experiments, the subjects in the experiment were not debriefed, given that such emails are very common in researchers’ everyday work and debriefing would likely pose a larger burden than benefit. A potential concern with audit studies similar to our paper is that if the fictitious student does not reply or behave untactfully in another way, and if their name or other characteristic are associated with a larger group, subjects in the experiment may become more likely to discriminate against future *real* candidates who are members of the group. We do not believe our study poses such a threat given that we used a neutral name that is not associated with any socio-demographic group (see below). Overall, the benefits of illuminating potential discrimination on career intentions that many individuals report being a source of substantial distress were judged important enough to conduct a study involving deception.

### 3.1. Participants

Emails and names of PIs were obtained from the Web of Science database accessed through the Collaborative Archive & Data Research Environment (CADRE) platform of Indiana University in November of 2022. First, we identified a set of journals core to the fields of microbiology and immunology (e.g., *Immunity* and *Nature Immunology*, see Table S1, Supplementary Appendix for the complete list). The journals were chosen by a domain expert (2nd author) using the following criteria: journals needed to be focused on microbiology and immunology, focused on basic rather than clinical research to increase the likelihood that the authors’ institutions had a PhD program, and span a range of impact factors. We then used Web of Science to identify all articles published^3^ in those journals between 2016 and 2021.

We collected the emails of the corresponding authors who were assumed to be the PIs as per fields’ norms. To narrow the scope of the experiment to U.S. institutions, we excluded email addresses that did not contain “.edu” at the end. Duplicates and University of Michigan emails were removed.

We conducted a power analysis to estimate the number of participants needed for each treatment condition. We hypothesized a response rate of 20 % for the industry condition and 25 % for the academia condition. For a two-sided test of proportions to have 0.8 power to detect such a difference at *α* = 0.05, each condition needs 1094 observations, and the number needed for a one-sided test falls to 862. Consequently, we aimed for 1000 subjects per treatment condition, and assigned any additional available emails to the Control group.

The final list contained 2442 unique email addresses. These emails were randomized into the following conditions: *control* (T0) with 442 participants, *industry* (T1) with 1000 participants, and *academia* (T2) with 1000 participants. The email was sent by a (fictitious) prospective student applying to PhD programs and interested in the work and potential mentorship of the PI. The student was identified as Jamie Anderson, a name chosen to be ambiguous and not be connected to any socio-demographic group. The student was portrayed as a senior Bachelor’s student. We made this choice because it is common for students in the U.S. to go straight from the Bachelor’s to a U.S.-based PhD program. Additionally, the student had two years of research experience, which may substitute somewhat for a Master’s degree in the eyes of mentors. The 2442 emails were sent from a University of Michigan email account linked to the fictitious student using the software “Yet Another Mail Merger” (YAMM) on December 13 and 14 of 2022.

### 3.2. Experimental conditions

**Fig. 2.**
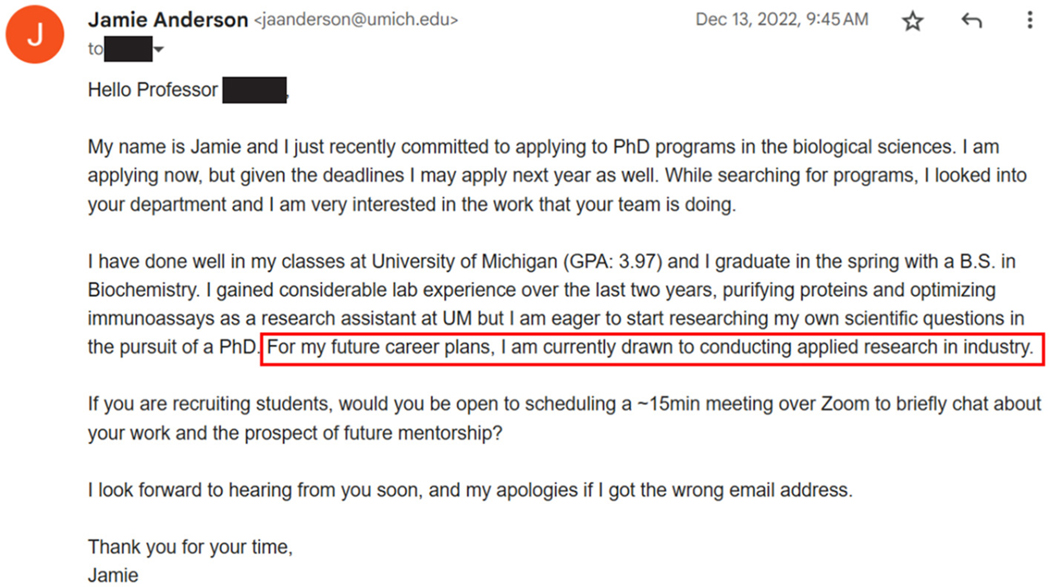
The email that was sent to experiment’s participants in the *industry* condition T1. The red box indicates the sentence that was variable between each experimental condition. (For interpretation of the references to color in this figure legend, the reader is referred to the web version of this article.)

We compare three different conditions, where we varied student’s indicating their future career interests. We provide an illustration of the email that was distributed in the *industry* condition T1 in Fig. 2. Condition T2 used the identical email except using “*For my future career plans, I am currently drawn to conducting basic research in academia.*” instead of the industry career goals. The control condition T0 did not include the sentence about future career plans at all. The subject line “*Prospective PhD student interested in your work*” was identical for each condition.

We finished data collection on February 20, 2023 (approximately two months after the email send-out). The YAMM software allowed us to track whether emails were successfully sent and opened. Of the 2442 emails that were sent, 1398 emails were opened, 240 emails were bounced and returned with an error message, and 804 emails remained unopened. For our analyses, we focused on the emails that were opened by the participants (corresponds to 57 % of the original sample) as our experimental manipulation was situated inside the email’s text.

To ensure that the recipients of the excluded emails are not systematically different from the participants we focus on, in Table S4 (see Supplementary Appendix) we report summary statistics for participants’ characteristics for the emails that were excluded from our main analysis. Performing pairwise *t*-test mean comparisons between the included and excluded emails, we found that our group of included respondents had the same level of industry connections as the recipients of the excluded emails, had longer careers (1.8 years difference in career length, *p-*value = 0.000; *t*-statistic = 3.787), and were affiliated with institutions of a slightly lower rank (3.2 points difference in score, *p-*value = 0.011; *t*-statistic = −2.562). The differences, however, are only marginal, and we believe they do not bias our results. For robustness, we also repeat our main analysis on the full sample of all sent-out and non-bounced emails (Table S5, see Supplementary Appendix). The covariate comparisons and regression results corroborate our findings for the opened emails.

### 3.3. Variables

#### 3.3.1. Focal variables

##### 3.3.1.1. Response.

Our main outcome variable is the indicator *Response*, set to 1 if the participant replied to the email and 0 otherwise. Among the 1398 opened emails in our data, 805 participants (58 %) responded. Among the respondents, 310 were to the emails from the Industry condition, 327 from the Academia condition, and 168 from the Control condition.

##### 3.3.1.2. Condition.

We used a categorical variable *Group* that was coded as Control (*n* = 285), Industry (*n* = 568), or Academia (*n* = 545) for our three experimental conditions.

#### 3.3.2. Moderators

To test moderation hypotheses H2-H4, we supplemented the data on authors from Web of Science with data from two other databases. First, we used DOIs to match papers in our sample to *OpenAlex* (accessed in May 2023)—one of the largest and widely used bibliometric databases (Singh Chawla, 2022). We were able to link 1395 (99.8 %) papers to *OpenAlex* and obtain author IDs. For each author we retrieved information about their current and previously recorded affiliations, their publication list, and co-author list.

To test H2, we computed an indicator *Industry connections* for each author in the sample based on the type of their affiliation as categorized by *OpenAlex*. We set the variable to 1 if an individual had an affiliation whose type was “company” (“education” was the most common affiliation type) currently or in the previous years. When accounting for current and all previous affiliations, 662 (47 %) researchers are currently or were at some point in their career affiliated with a company. Among those, 17 participants had a current affiliation with a company along other affiliations they possess (9 of these individuals responded to our email). For robustness, in Table S6 (see Supplementary Appendix) we excluded these individuals and repeated our analyses—our findings hold.

To test H3a and H3b, we computed the variable *Seniority* measured as the number of years passed since the PI’s first publication year. We were able to gather this information for 1260 participants (90 %) in our sample. We excluded from analysis the 10 % of records where *Seniority* was over 50 years on the assumption that these records were wrongly recorded by *OpenAlex*.

To test H4, for each PI, we measured the prestige of their institutions by linking their current affiliation to the *QS World University Rankings* report for the year of 2023. The QS ranking system captures an institution’s reputation and research performance (Selten et al., 2020). The report contained an overall score for almost 1500 institutions worldwide that was based on a set of criteria from academic reputation to the number of international students enrolled with a maximum score of 100. For matching, we used the Research Organization Registry (ROR) API with a required similarity score of at least 90 %.^4^ We first matched all institutions from the QS ranking table to their corresponding ROR IDs, then matched all affiliations of PIs in our sample to their ROR IDs, and finally joined the two datasets on ROR ID. We were able to compute the *Institutional prestige* for 733 PIs (52.4 %). Some affiliations were not matched to a QS record because either the ROR API call returned a null result or the record was not among the QS records matched to ROR IDs.

Table S2 (see Supplementary Appendix) provides summary statistics for the variables and their correlations. 47 % of the participants had industry connections. The participants’ seniority varied from 3 to 50 years since their first publication, with an average of 28 years that have passed. The participants’ institutions also varied greatly in rank, with an average QS Institutional prestige score of 60. A researcher’s seniority and industry connections are moderately correlated (*r* = 0.32).

Table S3 (see Supplementary Appendix) provides summary statistics for the variables by experimental condition. We also performed pairwise *t*-test mean comparisons for our key variables across conditions. We found that participants in our *industry* vs. *academia* groups do not statistically differ from each other in terms of *industry connections* and *seniority* (*p*-value >0.1 in both cases). The participants in the *industry* group seem to be affiliated with slightly more prestigious institutions compared to the PIs in the *academia* condition, but the difference is marginal (3.6 points difference in score) and only weakly significant (*p*-value = 0.051; *t*-statistic = 1.952). We thus conclude that our two key experimental groups are balanced in terms of participants’ observable characteristics. For robustness, we control for these observables in our regression models presented below.

## 4. Results

### 4.1. Content of replies

First, we performed a qualitative analysis of a sample of replies. We selected at random 20 % of replies from each experimental condition, which corresponded to 34 replies from the *control* group, 62 replies from *industry*, and 66 from *academia*. Each email had an equal probability of being included in the sample, regardless of the email respondent’s name, institution, status, seniority, or the response date/time. We then hand-coded each response as being 1) positive, 2) negative, 3) requesting more information from the student (i.e., their resume or research publications), or 4) other. In the *control* condition, 38 % of the responses were positive, i.e., a PI agreed to talk to the student. Whereas within both *industry* and *academia* groups, this rate was even higher: more than half of all replies were positive. In *industry*, 37 replies (60 %) were positive while only 12 PIs (19 %) declined the chat request often stating that they were not currently recruiting students. Within *academia*, 37 replies (56 %) were positive and 16 (24 %) were negative. This qualitative coding suggests that responses had similar composition of content across the treatment conditions (and potentially even more positive sentiment in the *industry* group), supporting the use of *Response* as a binary variable.

### 4.2. Main effect

**Fig. 3.**
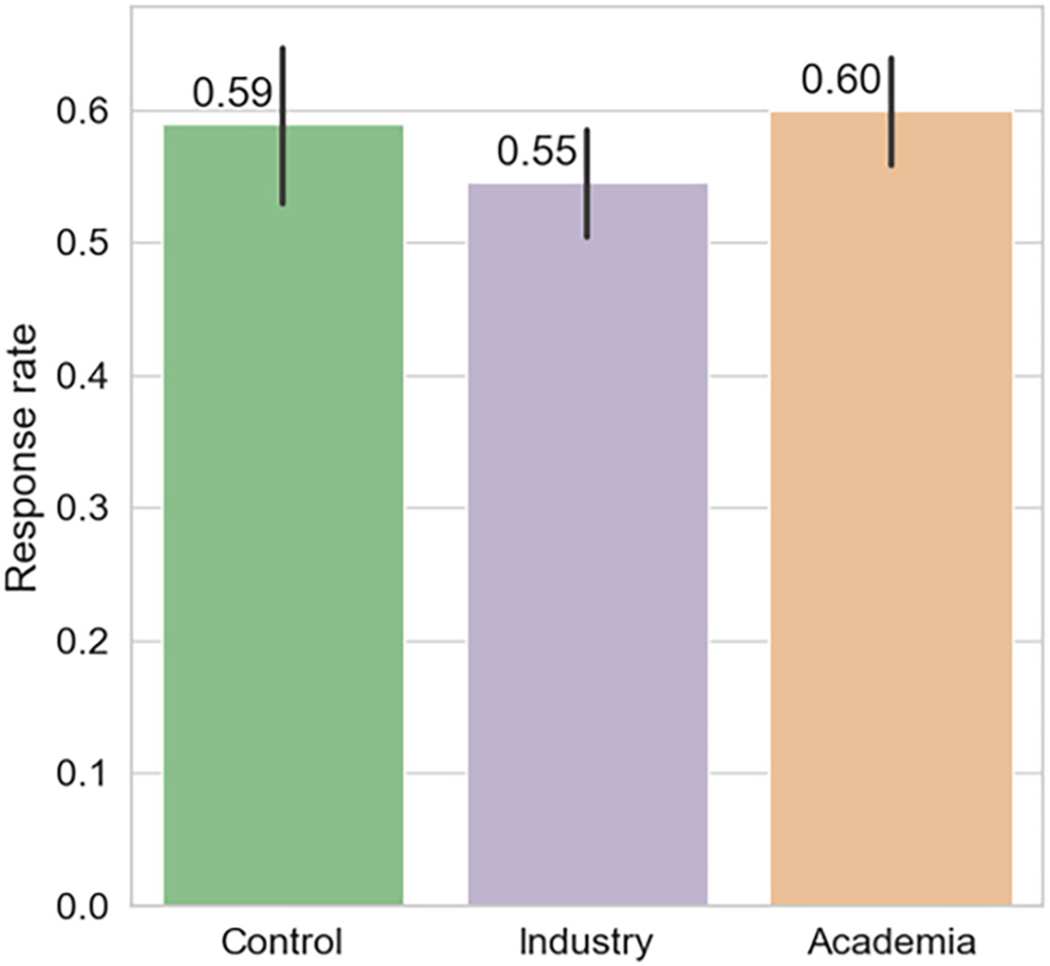
Response rate by treatment condition. Error bars represent 95 % CIs.

Next, we conducted mean comparisons between the experimental conditions, displayed in Fig. 3. The results of the *t*-test for the key comparison for H1 of *industry* vs. *academia* show the difference was small (—0.054) and not statistically significant (*p-*value = 0.068; *t*-statistic = —1.829). These first results provide at most suggestive evidence for H1.^5^

To test H1 further, we estimated a linear probability model of *Response* regressed on the focal variable *Group* and controls for journal and publication year. The results are displayed in Table 1, with Model 1 including *Group* only and Model 2 including covariates. Models 3–5 add each of the three moderator variables (focal researcher’s industry connections, seniority, and institutional prestige) separately, and Model 6 combines all controls and moderators together in one specification. Across all models, we found no robust support for H1, in line with the simple comparison of response ratios above.

### 4.3. Moderator: Industry connections

Next, we test H2. Examining the raw data, we found that our two treatment conditions are pretty similar in the average number of industry-affiliated researchers (Table S3, see Supplementary Appendix). Interestingly, the share of industry-connected individuals was 4 % higher in the academia (vs. industry) condition.

We then compared mean response rates across combinations of the *Industry connections* indicator and experimental conditions. Among PIs who have never had an explicit industry affiliation, replies favor academia-condition students but the difference is not statistically significant (coefficient = −0.036, *p*-value = 0.398; *t*-statistic = −0.847). Similarly, among PIs with industry connections, replies favor academia-condition students but the difference is not statistically significant (6.7 % higher response rate, *p*-value = 0.109; *t*-statistic = −1.607).

Table 2 reports regression analysis for our H2. We estimated a series of linear probability models that linked the *Response* indicator to experimental condition indicators, industry connections and its interaction with condition, experience, institutional prestige, journal, and publication year. We expected the interaction term between the *Industry* treatment and *Industry connections* to be positive. Contrary to expectations, the point estimate coefficient for the interaction term in Model 2 was actually negative (—0.038) and not statistically significant (*p*-value = 0.527). Model 3 adds other moderators as control variables and further supports this result. Overall, both regression and *t*-test analyses show no evidence that having industry connections is associated with higher response rates to industry- vs. academia-oriented students.

While *Industry connections* captures the PI’s direct connections to companies, they might also have some indirect industry connections through their co-authors. Accordingly, for each PI we computed the fraction of all-time co-authors who currently have industry affiliation. The Pearson correlation coefficient between *Industry connections* variable and this alternative measure is 0.21. Both industry and academia treatment groups had a small average share of company-connected co-authors, 3.5 % and 3.3 % respectively. Regressions using this alternative measure of proximity to industry are reported in Table S7 (see Supplementary Appendix). Results corroborate those from the main measure—we found no statistically significant evidence that industry proximity was associated with higher response rates (*p*-value >0.1).

### 4.4. Moderator: Seniority

We now turn to testing how PI’s level of seniority is associated with replies. Table S3 (see Supplementary Appendix) shows that the PI’s average career length since first publication is similar across experimental conditions in the raw data.

Table 3 reports results for our regression analysis where we test the interaction effect between the *Industry* treatment and a researcher’s *Seniority*. In all specifications the effect of *Industry* was not statistically different from the reference group *Academia* and the main effect was not statistically significantly changed by *Seniority.* We thus cannot reject the null hypothesis that there is no clear association between a researcher’s *Seniority* and their response rates to students depending on their career interests.

### 4.5. Moderator: Institutional prestige

Next, we test whether PIs affiliated with more prestigious institutions respond at different rates across experimental conditions. Table S3 (see Supplementary Appendix) shows that the means of participants’ QS institution scores across experimental conditions are similar, although PIs assigned to *Industry* come from slightly more prestigious institutions, on average.

Table 4 shows the regression analysis for H4. The main effect of *Institutional prestige* was negative and weakly significant (*p*-value = 0.058 in Model 3 of Table 4), which is in line with our baseline expectation that PIs from higher-prestige institutions generally show lower response rates to chat requests regardless of the condition. Similar to other moderation effects, we found no support for our hypothesis regarding institutional prestige. In Model 2 of Table 4, the interaction coefficient was essentially zero and highly insignificant (*p*-value = 0.984). We thus cannot reject the null that prestige is not associated with response rates across conditions.

## 5. Discussion

### 5.1. Summary of findings

Prior work and anecdotal evidence from academia have documented an association between trainees’ career intentions and mentors’ support, such that when trainees seek non-academic careers, mentors offer less support. Trainees who receive less support often experience psychological distress as a result. However, prior work has not established whether the association between trainees’ career intentions and mentors’ support is causal. This paper provides the first experimental evidence by using an audit experiment in the life sciences. We examined responses from 2442 life sciences PIs to prospective PhD students expressing career interest in industry, academia, or no mention (control). Results showed that PIs responded equally across all career interests, contradicting previous assumptions theorized in H1. No response rate differences were found based on PI’s industry connections, career length, and institutional prestige (H2 through H4), suggesting no discrimination against trainees with industry career intentions.

### 5.2. Potential explanations of the null result

What might explain this surprising null result? We consider several possibilities. First, the association between career intentions and mentorship may be causal but only when the trainee’s human capital is ambiguous—in such cases mentors may use career intentions as a signal of human capital, particularly commitment. In our design, however, human capital was relatively unambiguous—the emails in all conditions described the student as highly skilled and appropriately experienced. An intriguing follow-up study would be to replicate our experiment *without* signaling high human capital.

Second, the association may exist but not be causal. For example, it might be due to reverse causation: if PIs provide less mentorship to particular trainees, those trainees may choose to pursue different careers, not the other way around.

Third, the association may simply not exist. Such could be if the presumed benefits of same-career trainees are overstated. Recall that, in our theoretical framework, career intentions were predicted to causally affect mentorship because intentions a) signal to the mentor the trainee’s human capital and b) (long-term) benefits in terms of social capital, as well as c) threaten the mentor’s self-concept and raise doubts about their ability to mentor students for careers in other industries. The mechanism (b) drew on evidence that PIs benefit from a “sales force” to promote their work (Azoulay et al., 2019), which often consists of graduate students and other trainees who stay in academia and keep promoting their mentor’s work even long after the training period. Yet such benefits might be offset by industry-related ones: placing trainees into industry may help mentors access resources available (only) outside of academia. We argued that this mechanism requires trainees to graduate and build careers outside academia, delaying realized benefits for mentors and thus be of second-order consideration when making selection decisions. We acknowledge the possibility that this mechanism may be more important in reality than theorized, particularly in the capital-intensive areas of the life sciences, where industrial labs may have more or different equipment and materials.

Finally, the null result may be due to our research choices. For example, it is possible that the treatment was of too low an intensity to be salient. Although this possibility remains, similar audit experiments also administered via emails reported substantial treatment effects (Ajzenman et al., 2023; Buzard et al., 2023; Milkman et al., 2015; Rivera and Tilcsik, 2023). Additionally, PIs may view career intentions at the early, prior-to-application stage as not informative of future choices and not discriminate based on them.

### 5.3. Implications

Our study contributes to the extensive work on scientific labor markets and organizational decision making more broadly. Existing research on scientific labor markets has focused primarily on faculty hiring in their quest to explain what drives inequalities in academic careers (Clauset et al., 2015; Hofstra et al., 2022; Samble, 2008), typically considering applicant demographic characteristics (Moss-Racusin et al., 2012; Rivera, 2017). What has been opaque are objectives employers may have that arise from the interdependencies between employees’ careers and their own. In the domain of mentorship, these translate to the objective of having the trainees stay in the same industry for a long time. Our findings paint an arguably positive picture—in the academic life sciences, mentors do not discriminate against industry-leaning prospective PhD students. This is good news for PhD candidates who would like to or need to get PhD training but are unsure about or uninterested in staying in academia long-term.

Conceptually, our work challenges the widespread assumption, based on surveys and anecdotal evidence, that career mismatches *cause* lower mentorship. And because the causal relationship is challenged, so are the three mechanisms—human capital, social capital, and mentor’s self-concept—hypothesized to drive it. First, it is possible that the human capital considerations are the primary driver of the association—in our design, the human capital signal was not ambiguous, thus leaving the question on the strength of this mechanism in guiding selection decisions unanswered. Second, the social capital benefits of trainees staying in the same industry might be overstated and/or multifaceted with the potential benefits coming from a trainee bridging mentor with a different industry and its resources more important than hypothesized. Lastly and related, our null finding challenges, but certainly not conclusively disproves, the mechanism that a trainee’s same-industry career is in the mentor’s self-interest, and that this self-interest is substantial in their decision-making when selecting trainees. More research on testing the exact mechanisms and their interplay is needed.

Beyond implications for empirics and theory, our findings contribute to the recent policy calls to transform the PhD training model to meet the changing labor market demands (Editorial., 2023; Ganguli et al., 2022; Sauermann and Roach, 2016). In recent decades there has been a steady increase in the number of PhDs created by U.S. institutions (Cyranoski et al., 2011; Ghaffarzadegan et al., 2015), outpacing academic job availability and forcing many qualified individuals to either put increasingly more time into lower-wage postdoc and adjunct positions or to find work outside the academy (Ashenfelter and Card, 2002; Ghaffarzadegan et al., 2015; Opsomer et al., 2021). As a result, non-academic career paths became more of an industry norm rather than an exception, with many policy calls to train faculty to account for the reality of students’ career prospects and adjust the mentorship and resources provided to trainees to set them for the path to success in a diverse job market (e.g., Bosch and Casadevall, 2017; Lenzi et al., 2020; Leshner, 2015). The failure to do so reinforces a system that sets a significant number of trainees up for psychological distress and does not acknowledge the realities of the mentors’ labor market and attractive options outside of it (Marin and Vona, 2023). Our study suggests that the picture might not be as grim as assumed, and, if so, resources invested into mentor trainings might be better spent elsewhere.

### 5.4. Limitations and future work

Our study bears several limitations. First, our analysis focuses on only some potential determinants of mentors’ preferences for trainees. For example, mentors’ past experiences with industry-oriented trainees, their current research funding sources, and their own career trajectories could all influence their preferences for what students they would like to mentor, and are important to pursue in future work. Second, our treatment’s wording—“drawn to…”—prioritized external validity over treatment’s strength. This treatment may have ended up weak and/or gone unnoticed by many participants. Third, our design focuses on the early, pre-application stage in the training pipeline. These early stages have received very little scholarly attention despite their tremendous impact on the composition of professions and organizations (Lane et al., 2023; Milkman et al., 2015). Consequently, to develop our theoretical framework, we drew on the broader literature analyzing post-entry stages. Employers’ criteria or even decision-making process may vary in the earlier vs. later stages. Nevertheless, our work has value in revealing this discrepancy, opening an important avenue of inquiry. Fourth, our measure of *Industry connections* via affiliations and coauthorship ties, used to test H2, might not meaningfully proxy mentors’ *attitudes* towards industry careers. In the field of life sciences, the research often requires industry connections for equipment and access to experimental settings, as well as additional funds for larger-scale studies. Industry connections may thus have more to do with the PIs’ “need” to collaborate with industry than their favorability of industry careers. Future research should consider alternative and more direct ways of measuring attitudes. Additionally, our operationalization of mentor seniority and institutional prestige are not the only plausible ones. For instance, seniority could be measured with position rather than years since the first publication, and prestige with departmental rather than university rankings. Lastly, our sample of researchers was drawn from corresponding authors of publications in the prominent life sciences journals that disproportionately come from large, research-intensive institutions. As a result, the findings might not generalize to smaller and less research-intensive institutions.

Our study opens several avenues for future research. First, more evidence on the other stages of the PhD entry and training pipeline is needed, with the eventual goal of developing “right,” evidence-based mitigation strategies for reducing trainee distress. For example, mentors’ priorities can shift over time and their careers can themselves evolve towards having more industry ties, which might affect attitudes about placing students into industry. Second, our study only focuses on the life sciences, so extensions to other academic disciplines may be fruitful. For instance, mentor decision-making might vary with the PhD program’s funding model or lab size. Relatedly, our study examines graduate training in the U.S., while mentors in other countries might have differing attitudes towards industry careers and the PhD programs might even follow different training models (e.g., Enders, 2005). Third, varying our experimental set-up may yield benefits. For example, our emails used relatively unambiguous signals of high human capital. Avoiding such strong and direct signals may reveal that career intentions signal human capital, revealing the main mechanism driving a causal effect. Finally, future research relying on qualitative interviews with PIs and advanced behavioral research methodologies like eye-tracking would help give in-depth insights into the motivations behind mentors’ selection decisions and why they decide to respond or not to an incoming email request.

In conclusion, this paper takes an important step towards illuminating a critical aspect of the scientific labor market, finding a surprising absence of discrimination against prospective mentees with industry career intentions.

## Supplementary Material

MMC1

Supplementary Appendix

Supplementary materials to this article can be found online at https://doi.org/10.1016/j.respol.2025.105232.

## Figures and Tables

**Table 1:** Regression analysis for H1.

Dependent variable: Responded yes/no						

	(1)	(2)	(3)	(4)	(5)	(6)

Group = Industry	−0.054* (0.030)	−0.056* (0.030)	−0.053* (0.030)	−0.044 (0.031)	−0.067 (0.041)	−0.033 (0.043)
Group = Control	−0.011 (0.036)	−0.012 (0.036)	−0.010 (0.036)	−0.003 (0.038)	0.041 (0.051)	0.052 (0.054)
Industry connections			0.036 (0.027)			−0.020 (0.042)
Seniority				0.004*** (0.001)		0.004* (0.002)
Institutional prestige					−0.002* (0.001)	−0.002** (0.001)
Intercept	0.600*** (0.021)	0.536*** (0.056)	0.514*** (0.059)	0.439*** (0.069)	0.635*** (0.099)	0.565*** (0.115)
Publication year controls	NO	YES	YES	YES	YES	YES
Journal controls	NO	YES	YES	YES	YES	YES
*N*	1398	1398	1395	1260	733	660
*Adjusted R* ^*2*^	0.001	0.003	0.004	0.013	0.012	0.020

*Note:* Standard errors are in parentheses. *Academia* group is the reference point for experimental group comparisons.

* *p* < 0.1

** *p* < 0.05

*** *p* < 0.01.

**Table 2:** Regression analysis for H2.

Dependent variable: Responded yes/no			

	(1)	(2)	(3)

Industry connections	0.037 (0.027)	0.084** (0.043)	0.058 (0.063)
Group = Industry		−0.034 (0.042)	−0.000 (0.058)
Group = Control		0.061 (0.049)	0.152** (0.069)
Industry × Industry connections		−0.038 (0.060)	−0.072 (0.086)
Control × Industry connections		−0.162** (0.073)	−0.253** (0.109)
Seniority			0.004* (0.002)
Institutional prestige			−0.002** (0.001)
Intercept	0.492*** (0.057)	0.483*** (0.062)	0.515*** (0.118)
Publication year controls	YES	YES	YES
Journal controls	YES	YES	YES
*N*	1395	1395	660
*Adjusted R* ^*2*^	0.003	0.006	0.025

*Note:* Standard errors are in parentheses. *Academia* group is the reference point for experimental group comparisons.

* *p* < 0.1.

** *p* < 0.05.

*** *p* < 0.01.

**Table 3:** Regression analysis for H3 (alternative H3a and H3b).

Dependent variable: Responded yes/no			

	(1)	(2)	(3)

Seniority	0.004*** (0.001)	0.003 (0.002)	0.003 (0.003)
Group = Industry		−0.099 (0.087)	−0.066 (0.123)
Group = Control		−0.045 (0.106)	0.029 (0.151)
Industry × Seniority		0.002 (0.003)	0.001 (0.004)
Control × Seniority		0.001 (0.003)	0.001 (0.005)
Industry connections			−0.020 (0.042)
Institutional prestige			−0.002** (0.001)
Intercept	0.421*** (0.067)	0.468*** (0.081)	0.584*** (0.132)
Publication year controls	YES	YES	YES
Journal controls	YES	YES	YES
*N*	1260	1260	660
*Adjusted R* ^*2*^	0.012	0.011	0.017

*Note:* Standard errors are in parentheses. *Academia* group is the reference point for experimental group comparisons.

* p < 0.1.

** p < 0.05.

*** p < 0.01.

**Table 4:** Regression analysis for H4.

Dependent variable: Responded yes/no			

	(1)	(2)	(3)

Institutional prestige	−0.002** (0.001)	−0.002 (0.001)	−0.003* (0.001)
Group = Industry		−0.068 (0.117)	−0.086 (0.124)
Group = Control		−0.031 (0.147)	−0.089 (0.155)
Industry × Institutional prestige		0.000 (0.002)	0.001 (0.002)
Control × Institutional prestige		0.001 (0.002)	0.002 (0.002)
Industry connections			−0.019 (0.042)
Seniority			0.004* (0.002)
Intercept	0.624*** (0.098)	0.648*** (0.114)	0.613*** (0.131)
Publication year controls	YES	YES	YES
Journal controls	YES	YES	YES
*N*	733	733	660
*Adjusted R* ^*2*^	0.008	0.010	0.018

*Note:* Standard errors are in parentheses. *Academia* group is the reference point for experimental group comparisons.

* p < 0.1.

** p < 0.05.

*** p < 0.01.

## Data Availability

The data that has been used is confidential.

## Footnotes

1 We thank an anonymous reviewer for pointing out that the social welfare implications of discrimination against trainees with mismatched career intentions are not straightforward. For example, it may be socially optimal for the costs of PhD training of trainees who go into industry to be borne by the hiring firm, and without a mechanism for such payments for the mismatches to be discouraged.

2 Microbiology is a broadly defined area that can encompass many other subfields of the life sciences. Microbiology departments are very common at academic institutions and are often paired with immunology because of a predominance of infectious disease research that happens in the biological sciences resulting from federal funding priorities. Broadening our approach to the life sciences generally would have resulted in having more clinician scientists in the sample who are comfortable with applied research, rarely mentor PhD students, and are less interested in scientific publishing, potentially biasing our results. We thus narrowed down our sample to microbiology and immunology specifically to ensure we target prospective PIs in academic institutions with PhD programs and reduce the number of clinicians in the sample.

3 The database at the time had incomplete coverage of 2021 publications.

4 See https://ror.org/blog/2019–11-25-untangling-affiliation-strings/ and https://ror.readme.io/docs/match-organization-names-to-ror-ids for more details. Accessed on July 25, 2023.

5 It is worth noting that the experiment was powered to detect a 5 % difference in reply rates, with the expectation that the average reply rate would be 0.225 (20 % vs. 25 %) and the difference between sent and opened emails would be small. Empirically, the response rate turned out to be 0.32 on average and the difference between sent and opened emails was substantial. A post-hoc analysis shows that, to detect the empirical 55 % vs. 60 % difference among opened emails given the empirical sample size of 1113 / 2 per group, the power is 0.62.
