# Supplementary material for: The effect of trainee career intentions on mentor’s interest in the trainee: Experimental evidence from academia
Source: Res Policy. Author manuscript; Available in PMC 2025 Jun 18. (PMC12176394; doi:10.1016/j.respol.2025.105232)
Supplement: MMC1 [file NIHMS2067685-supplement-MMC1.pdf]

# Supplementary Appendix

**Table S1**. Journals used for selection of study participants (n = 2,442).

| Journal Name | Journal Impact Factor (2021) | Number of Emails Collected |
|---|---|---|
| Immunity | 31.75 | 266 |
| Nature Immunology | 31.25 | 187 |
| Lancet Infectious Diseases | 21.37 | 186 |
| Trends in Immunology | 16.68 | 128 |
| Trends in Microbiology | 11.16 | 106 |
| PLoS Pathogens | 7.01 | 791 |
| mBio | 6.78 | 778 |

**Table S2**. Descriptive statistics and correlations for the sample of opened emails that were used for our main analysis (n = 1,398).

| Variable | *N* | Mean | S.D. | Min | Max | 1 | 2 | 3 |
|---|---|---|---|---|---|---|---|---|
| 1 Response (yes/no) | 1,398 | 0.58 (n = 805) | — | 0 | 1 | | | |
| 2 Industry connections | 1,395 | 0.47 | — | 0 | 1 | -0.01 | | |
| 3 Seniority | 1,260 | 28.3 | 10.91 | 3 | 50 | 0.09 | 0.32 | |
| 4 Institutional prestige | 733 | 60.07 | 22.38 | 24.3 | 100 | -0.09 | 0.13 | 0.07 |

**Table S3**. Summary statistics for the sample of opened emails that were used for our main analysis (n = 1,398) broken down by experimental condition.

| Variable | *N* | Mean | S.D. | Min | Max |
|---|---|---|---|---|---|
| | | Group = *Industry* | | | |
| Response (yes/no) | 568 | 0.55 (n = 310) | — | 0 | 1 |
| Industry connections | 568 | 0.47 | — | 0 | 1 |
| Seniority | 508 | 28.1 | 10.69 | 3 | 50 |
| Institutional prestige | 297 | 61.41 | 22.31 | 24.4 | 100 |
| | | Group = *Academia* | | | |
| Response (yes/no) | 545 | 0.6 (n = 327) | — | 0 | 1 |
| Industry connections | 544 | 0.51 | — | 0 | 1 |
| Seniority | 493 | 28.01 | 11.07 | 3 | 50 |
| Institutional prestige | 295 | 57.84 | 22.12 | 24.3 | 100 |
| | | Group = *Control* | | | |
| Response (yes/no) | 285 | 0.59 (n = 168) | — | 0 | 1 |
| Industry connections | 283 | 0.41 | — | 0 | 1 |
| Seniority | 259 | 29.26 | 11 | 3 | 50 |
| Institutional prestige | 141 | 61.89 | 22.82 | 26.1 | 100 |

**Table S4**. Summary statistics for participants' characteristics for the emails that were excluded from our main analysis.

| **Variable** | ***N*** | **Mean** | **S.D.** | **Min** | **Max** |
|---|---|---|---|---|---|
| *All emails that were excluded* | | | | | |
| Industry connections | 1,038 | 0.47 | — | 0 | 1 |
| Seniority | 958 | 26.53*** | 10.99 | 3 | 50 |
| Institutional prestige | 529 | 63.3** | 21.77 | 24.3 | 100 |
| *Broken down by the reason of exclusion:* | | | | | |
| *1. Bounced emails with a returned error message* | | | | | |
| Industry connections | 239 | 0.41* | — | 0 | 1 |
| Seniority | 219 | 23.38*** | 11.12 | 3 | 50 |
| Institutional prestige | 103 | 60.21 | 22.93 | 25.2 | 100 |
| *2. Unopened emails* | | | | | |
| Industry connections | 799 | 0.49 | — | 0 | 1 |
| Seniority | 739 | 27.46* | 10.79 | 3 | 50 |
| Institutional prestige | 426 | 64.05*** | 21.44 | 24.3 | 100 |

*Note:* We performed *t*-tests to compare the emails that were excluded from the analysis with the main sample of the emails that were analyzed. We performed pairwise comparisons for all excluded emails, as well as each of the subsamples within them (bounced and unopened emails). $^{*}$p<0.1; $^{**}$p<0.05; $^{***}$p<0.01 indicate whether differences between means were statistically significant.

**Table S5**. Regression analysis for H1—robustness test on the sample of all sent-out (including both opened and unopened) and non-bounced emails.

| **Dependent variable: Responded yes/no** | (1) | (2) | (3) |
|---|---|---|---|
| Group = Industry | -0.019 | -0.023 | -0.029 |
| | (0.023) | (0.023) | (0.032) |
| Group = Control | 0.063$^{**}$ | 0.057$^{*}$ | 0.063 |
| | (0.029) | (0.029) | (0.042) |
| Industry connections | | | -0.030 |
| | | | (0.031) |
| Seniority | | | 0.003$^{**}$ |
| | | | (0.001) |
| Institutional prestige | | | -0.002$^{***}$ |
| | | | (0.001) |
| Intercept | 0.362$^{***}$ | 0.307$^{***}$ | 0.374$^{***}$ |
| | (0.016) | (0.042) | (0.086) |
| Publication year controls | NO | YES | YES |
| Journal controls | NO | YES | YES |
| *N* | 2,202 | 2,202 | 1,056 |
| *Adjusted* $R^2$ | 0.003 | 0.009 | 0.028 |

*Note:* $^{*}p<0.1$; $^{**}p<0.05$; $^{***}p<0.01$. Standard errors are in parentheses. *Academia* group is a reference point for experimental group comparisons.

**Table S6**. Regression analysis for H1-H4—robustness test on the sample of opened emails excluding 17 participants with current company affiliation.

| **Dependent variable: Responded yes/no** | | | | | | |
|---|---|---|---|---|---|---|
| | (1) | (2) | (3) | (4) | (5) | (6) |
| Group = Industry | -0.053* | -0.054* | -0.033 | -0.000 | -0.066 | -0.086 |
| | (0.030) | (0.030) | (0.043) | (0.058) | (0.123) | (0.124) |
| Group = Control | -0.002 | -0.003 | 0.052 | 0.152** | 0.029 | -0.089 |
| | (0.036) | (0.036) | (0.054) | (0.069) | (0.151) | (0.155) |
| Industry connections | | | -0.020 | 0.058 | -0.020 | -0.019 |
| | | | (0.042) | (0.063) | (0.042) | (0.042) |
| Seniority | | | 0.004* | 0.004* | 0.003 | 0.004* |
| | | | (0.002) | (0.002) | (0.003) | (0.002) |
| Institutional prestige | | | -0.002** | -0.002** | -0.002** | -0.003* |
| | | | (0.001) | (0.001) | (0.001) | (0.001) |
| Industry X Industry connections | | | | -0.072 | | |
| | | | | (0.086) | | |
| Control X Industry connections | | | | -0.253** | | |
| | | | | (0.109) | | |
| Industry X Seniority | | | | | 0.001 | |
| | | | | | (0.004) | |
| Control X Seniority | | | | | 0.001 | |
| | | | | | (0.005) | |
| Industry X Institutional prestige | | | | | | 0.001 |
| | | | | | | (0.002) |
| Control X Institutional prestige | | | | | | 0.002 |
| | | | | | | (0.002) |
| Intercept | 0.598*** | 0.541*** | 0.565*** | 0.515*** | 0.584*** | 0.613*** |
| | (0.021) | (0.057) | (0.115) | (0.118) | (0.132) | (0.131) |
| Publication year controls | NO | YES | YES | YES | YES | YES |
| Journal controls | NO | YES | YES | YES | YES | YES |
| *N* | 1,381 | 1,381 | 660 | 660 | 660 | 660 |
| *Adjusted* $R^2$ | 0.001 | 0.003 | 0.020 | 0.025 | 0.017 | 0.018 |

*Note:* *p<0.1; **p<0.05; ***p<0.01. Standard errors are in parentheses. *Academia* group is a reference point for experimental group comparisons. Models 1-3 test our H1, Model 4 tests H2, Model 5 tests H3 (alternative H3a and H3b), and Model 6 tests H4.

**Table S7**. Regression analysis for H2—robustness test using *fraction of industry-affiliated co-authors* as an alternative operationalization for the *industry connections* variable.

| **Dependent variable: Responded yes/no** | | | |
|---|---|---|---|
| | (1) | (2) | (3) |
| Fraction of industry-affiliated co-authors (%) | 0.002 | 0.000 | 0.009 |
| | (0.005) | (0.009) | (0.014) |
| Group = Industry | | -0.071 | -0.016 |
| | | (0.049) | (0.070) |
| Group = Control | | -0.001 | 0.070 |
| | | (0.062) | (0.092) |
| Industry X Fraction of industry-affiliated co-authors (%) | | 0.005 | -0.005 |
| | | (0.012) | (0.017) |
| Control X Fraction of industry-affiliated co-authors (%) | | -0.004 | -0.005 |
| | | (0.016) | (0.024) |
| Industry connections | | | -0.023 |
| | | | (0.043) |
| Seniority | | | $0.004^{*}$ |
| | | | (0.002) |
| Institutional prestige | | | $-0.002^{**}$ |
| | | | (0.001) |
| Intercept | $0.509^{***}$ | $0.538^{***}$ | $0.546^{***}$ |
| | (0.058) | (0.065) | (0.122) |
| Publication year controls | YES | YES | YES |
| Journal controls | YES | YES | YES |
| *N* | 1,395 | 1,395 | 660 |
| *Adjusted* $R^2$ | 0.001 | 0.001 | 0.016 |

*Note:* $^{*}$p<0.1; $^{**}$p<0.05; $^{***}$p<0.01. Standard errors are in parentheses. *Academia* group is a reference point for experimental group comparisons.
